# Advances in knowledge and practice benefiting the health and management of first permanent molars in children

**DOI:** 10.1038/s41415-024-8012-5

**Published:** 2025-01-24

**Authors:** Greig D. Taylor, Victoria Bulmer

**Affiliations:** 41415278286001 School of Dental Sciences, Newcastle University, Newcastle, UK; Population Health Sciences Institute, Newcastle University, Newcastle, UK; 41415278286002 School of Dental Sciences, Newcastle University, Newcastle, UK

## Abstract

First permanent molars (FPMs) remain the most affected teeth by dental conditions in childhood. Maintaining the health of FPMs should be prioritised by dental professionals. However, if subjected to unfavourable circumstances, FPMs can become compromised and impact the child negatively. In this article, we highlight current thinking and provide practical tips to prevent FPMs from becoming compromised. The importance of including the young person in decision-making and the influence different dental conditions might have on management of FPMs are discussed. Finally, the impact and treatment options available for FPMs should they become compromised are explored, focusing on the main question of whether to restore or extract these teeth.

## Key points

- Prevention of disease in first permanent molars should be a key focus of providing dental care for any child patient.
- Young people should be actively involved in shared decisions for dental care, ensuring their autonomy is being respected.
- Professionals need to consider the relevant patient-, mouth- and tooth-level factors, as well as patient and parent views and preferences to ascertain how best to manage the compromised first permanent molar for that individual.

## Introduction

Promoting and maintaining the health of the first permanent molar (FPM) is key for any child. FPM teeth begin to erupt around the age of six years; however, embryonic development begins around the twentieth week *in utero* with hard tissue formation occurring some 8-12 weeks later. The foundation of optimal dental health is grounded in evidence-based prevention.^1^ Concerted efforts should be made by dental providers to prevent and/or arrest disease in FPMs, especially given the negative impacts to the child, their families, the health system and society, should they become compromised and need an intervention.^2^^,^^3^^,^^4^

In this article, we initially provide an update on the disease burden and impact of FPMs, before discussing how to prevent disease in FPMs. Thereafter, we focus on the importance of including the child in shared decision-making for FPMs, the influence different dental conditions might have on management, and treatment options for FPMs as they become compromised.

## The ‘compromised' first permanent molar - why the fuss?

If subjected to unfavourable circumstances, FPMs can become compromised, and can therefore be referred to as a compromised first permanent molar (cFPM). There are many causes of cFPMs; however, dental caries and/or the developmental anomaly molar-incisor hypomineralisation (MIH) are often regarded as the most common.

### Dental caries

It is well-accepted that the FPM is the most susceptible permanent tooth to dental caries, with evidence from cross-sectional,^5^^,^^6^ short-term^7^ and long-term observational and life-course studies^8^ confirming this. In the United Kingdom (UK), the most recent Children's Dental Health Survey (England, Wales, and Northern Ireland) in 2013 reported that by age 15, 25% of FPM teeth will have obvious dental caries experience compared to the next most prevalent permanent tooth - the second permanent molar - at 9%.^5^ Morphological variations of FPM compared to other permanent teeth are likely to contribute to this increased susceptibility to dental caries.^6^^,^^9^ An alternative explanation could be that these teeth are the first permanent teeth to erupt and therefore spend the longest time in the mouth. Although anecdotal, the additional years of being exposed to mediators of the biofilm may contribute to the increased susceptibility of these teeth to dental caries in comparison to adjacent permanent teeth.

### Molar-incisor hypomineralisation

The susceptibility to dental caries is further increased if the FPM is developmentally abnormal, most commonly by a condition known as MIH.^10^ MIH is qualitative enamel defect that leads to a reduction in the mineral content of enamel of FPMs and occasionally incisors.^11^ A recent meta-analysis reported several peri- and post-natal factors during early childhood were associated with increased chance of developing MIH, whereas pre-natal factors were not.^12^ Despite this, the exact causative mechanisms of MIH have yet to be established;^13^ although, recent evidence supports the idea that epigenetic changes in the genome, due to the influence of environmental factors, could be the most likely explainable mechanism.^14^ Therefore, the timing of these environmental insults support the theory as to why FPMs are vulnerable to being hypomineralised.

It is reported that 27.4% of children diagnosed with MIH will need a clinical intervention.^15^ This is due to the hypomineralised molar having an inherent lack of structural integrity,^16^ which, compounded by normal biting forces, causes further mechanical destruction, known as post-eruptive breakdown. This affects the macroscopic appearance of these teeth which: a) makes them harder to treat; and b) increases their susceptibility to dental caries due to greater plaque accumulation^17^ and bacterial penetration.^18^

## If they become compromised, what is the impact?

A cFPM is likely to impact the child directly, as well as their families, the health system and society indirectly. Direct impacts associated with cFPMs are expected to have an intermittent effect throughout life and not just in childhood.^19^ Dental pain, in the form of irreversible pulpitis, from a tooth affected by caries will negatively cause impairment to a child's oral-health related quality of life (OHRQoL).^5^^,^^20^ More specifically, children and adolescents with cFPMs report issues with eating, sleeping and reduction in taking part in daily activities, as well as worse oral health experience and poorer school performance and attendance than caries-free children.^19^^,^^21^

Similarly, children with cFPMs due to MIH are likely to have a negatively impaired OHRQoL compared to children without MIH.^22^ It has been reported that 27.4% (95% CI: 23.5% − 31.7%) of children affected by MIH were, or will be in need of, treatment due to pain, sensitivity and post-eruptive breakdown.^15^ Compared to unaffected controls, those with MIH are approximately ten times more likely have undergone dental treatment on their FPM, with each affected tooth likely to have been treated, on average, twice.^15^

Indirectly, the families, healthcare systems and society can be impacted. Parents of children with cFPMs are known to have to take time off work to deal with impacts and consequences from these teeth.^19^ Although a specific figure is difficult to consider, the impact a diseased cFPM has on potential economic growth as a loss of productivity due to depletion in labour, capital and other production costs is likely.^4^ In addition, dental extractions, remains the most common reason for admission to hospital for children aged 5-9 years old (more than double the second most common condition, acute tonsillitis).^23^ The financial impact of, for the most part, a preventable dental condition, is a significant proportion of the NHS dental budget.^23^

Despite the prevalence and impact of cFPMs due to dental caries and/or MIH, for most children, the FPM erupts in a state of health.

## The erupting FPM - a period of real vulnerability

As the FPM erupts, the overlying operculum provides a sheltered micro-niche for plaque to accumulate, inducing an ecological shift in the biofilm environment. If the biofilm remains undisturbed, due to inadequate toothbrushing technique or unsupported toothbrushing, the biofilm will mature over time. Ultimately, this will lead to a homeostatic imbalance of the plaque biofilm and the formation of a cavitated carious lesion.^24^ As discussed, in children who have MIH, this process can be worsened and accelerated by the lack of structural integrity of these hypomineralised molars.^16^ Stressing the importance of prevention and tailoring evidence-informed actions and behaviours, together with professional support, aims to sustain health of FPMs across the life course.^1^^,^^25^

### Oral hygiene/toothbrushing instruction

To mitigate against this eruption vulnerability of the FPM, adjusting prevention with an emphasis to focus on cleaning around this erupting tooth is advised. To reduce plaque accumulation seen on partially erupted teeth, dental professionals should teach children and parents to change the position of their toothbrush so it is held in a buccal-lingual direction instead of the conventional mesial-distal placement.^26^^,^^27^ Upon eruption, positioning of the toothbrush should be encouraged to revert to the accepted norm. The parent or carer should be encouraged to assist with toothbrushing, particularly during eruption and/or until the child has adequate understanding and manual dexterity to undertake this independently.^1^^,^^25^ Disclosing the plaque will further aid both the child and their parent/carer to understand where the issue lies and what is required for optimum plaque removal.

### Fluoride

In addition to disrupting the biofilm, regular exposure to fluoride maintains a concentration in the plaque biofilm that encourages remineralisation of the tooth surface. Various methods are available to deliver fluoride, including toothpaste, water, milk, mouthrinses, tooth gels and varnish, with those which can be incorporated into aspects of everyday living being far more likely to be effective.^1^^,^^25^ Optimally fluoridated toothpaste, based on caries risk status, remains one of the most effective methods to maintain health.^28^ In an ideal situation, this would be carried out twice daily in the family home, supervised by parents/carers; however, it is acknowledged that for many, this can be challenging. Financial pressures are shown to lead to hygiene poverty, limiting the ability of some parents to afford the basic products necessary to maintain oral hygiene.^29^ Supervised toothbrushing programmes can partly overcome these financial concerns and are known to be effective in reducing tooth decay and health inequalities in children; however, the provision of these currently varies across the UK.^30^

Fluoride varnish (5% sodium fluoride) can be applied to FPMs, tenaciously adhering to the tooth surface, providing a slow release of fluoride in these vulnerable areas over a period of time.^31^ The evidence is overwhelming to support its effectiveness in reducing decay.^32^^,^^33^ Despite not being as technique-sensitive as fissure sealants, it is recommended that drying the FPM first will increase adherence. Conventionally, application is carried out in dental clinics;^1^ however, application in education facilties^34^ or as part of community engagement approaches^35^ are increasing and can overcome the barriers experienced by some in trying to access regular care.

### Fissure sealants

**Fig. 1:**
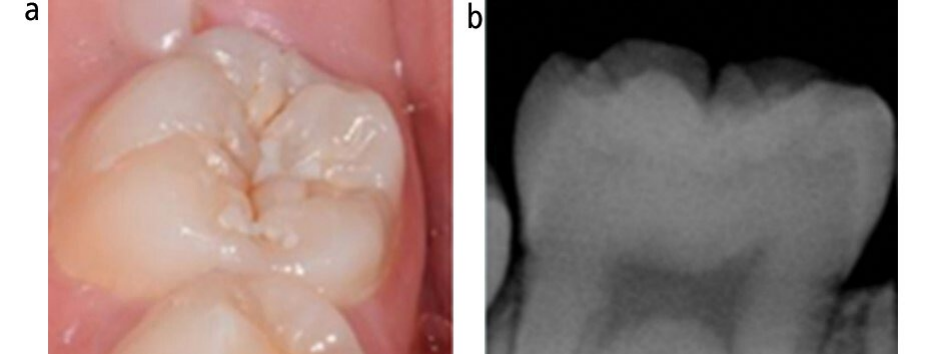
a, b) Tooth with microcavitation, suitable for a fissure sealant

In comparison to no sealant, the placement of a resin-based fissure sealant has been shown to reduce the absolute caries risk between 11% and 51% up to 48 months follow-up.^36^ Increased penetration and retention rates are observed when adhesive systems are used before applying a resin-based fissure sealant.^37^^,^^38^ Conventionally, fissure sealants are placed in non-cavitated FPMs of patients assigned a high-caries risk status^1^^,^^39^ **(**see Fig. 1**).** In recent years, however, this approach has been challenged. Cavitated enamel and microcavities in dentine carious lesions can be managed with a well-placed resin-bonded fissure sealant, with evidence suggesting their effectiveness at sealing in the caries and preventing any further progression.^38^^,^^39^ In these cases, prevention must be optimised and patients should have regular clinical and radiographic examinations to ensure any lesion progression is identified at the earliest time point. Similarly, resin-based fissure sealants should be placed on children with mildly affected hypomineralised FPMs as part of an MIH diagnosis.^10^ Strategies to enhance retention rates in hypomineralised molars involve extended etching or placement under rubber dam; although, the evidence to support these approaches is limited.^40^

It remains very clear that the evidence base confirms resin-based fissure sealants are superior to glass ionomer sealants in terms of their clinical effectiveness^36^ and therefore placing them should be the first-line approach. However, despite their effectiveness, a resin-based fissure sealant may not always be the most appropriate form of sealant to place. In children with limited compliance, or in circumstances where moisture control is compromised (eg partially erupting molar), a well-placed glass ionomer sealant could be used as an interim option, being replaced by a resin-based sealant at a later date.^26^

### Silver diamine fluoride

**Fig. 2:**
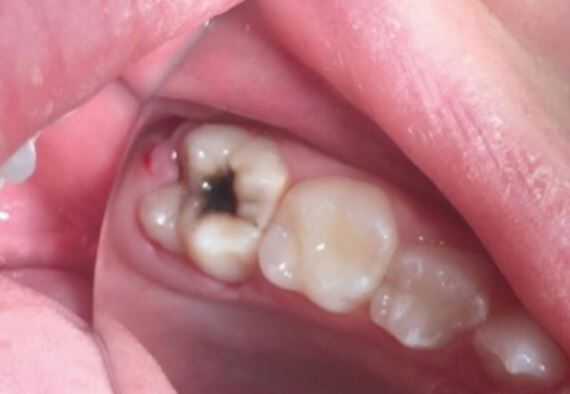
Application of SDF for a child who was not compliant to manage operative care in their FPM (hypomineralised)

Alternatively, it would be reasonable and feasible to consider applying silver diamine fluoride (SDF) in circumstances when an FPM has become cavitated and either the child is unable to co-operate for treatment (see Fig. 2) or the tooth is in a vulnerable position (eg partially erupted) that would not permit a restoration to be placed. In the UK, SDF is steadily becoming part of a dental professional's armamentarium to combat dental disease.^41^ Though its use specifically for FPMs currently remains anecdotal,^42^ its placement ‘buys' the dental professional some time, allowing definitive management strategies to be completed at a later date. Of course, there are risks associated with SDF placement; most notably, the black discoloration of the tooth **(**see Fig. 2), which should be discussed with patients in advance of its use.^41^ It is worth noting that, should the decision be made to definitively restore a FPM treated with SDF, then dental professionals should use a total-etch^43^ rather than a self-etching^44^ approach to overcome the known reduced bond strengths between composite and SDF-treated enamel and dentine.

### Public health measures

Maintaining the health of FPMs should not just focus on tooth- or patient-level interventions. Upstream public health approaches, such as implementing community water fluoridation schemes, supervised toothbrushing programmes and taxation of sugar-sweetened sugar beverages, aim to prevent disease at a population level. A recent review article highlights the benefits for children in implementing a community water fluoridation scheme.^45^ Despite opposition by some, as an upstream public health intervention, it is clinically effective,^46^ safe^47^^,^^48^ and indiscriminately delivers health benefits to a wide population.^45^

**Fig. 3:**
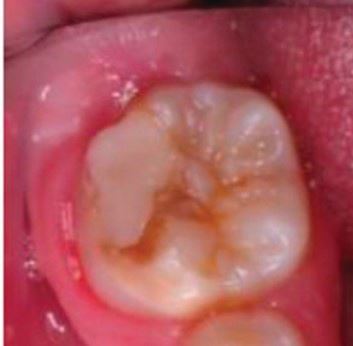
A compromised first permanent molar in a nine-year-old patient

Ultimately, adopting, implementing and encouraging a multi-pronged preventive approach should significantly reduce the risk of a young patient's FPM entering a lifelong cycle of needing restorative treatment. However, for a cohort of children, the FPM will be compromised and need a clinical intervention (see Fig. 3). Deciding what is best to do, though, can be challenging.^49^^,^^50^

## Shared decision-making - who should decide?

Whether trying to maintain health in a FPM, or deciding how to manage a cFPM, it is important that any decision is jointly made between the patient, parent and healthcare professional.^51^ Across healthcare, the extent to which healthcare providers involve patients in decision making is reported consistently as low.^52^ It is likely that professionals do not actively acknowledge or seek patient views, or that parents/carers and/or adolescents, are unsure of a shared decision-making concept.^53^When asked in a recent preference elicitation study, it appears the UK general public preferred to be wholly or partly involved in the decision-making process for cFPMs as opposed to the dentist making the decision alone.^54^A recent qualitative study with adolescents highlighted the importance of shared decision-making.^49^ The young people interviewed expressed a wish to assert their autonomy in any decisions made regarding cFPMs; however, a gap in perceived shared decision-making and actively involving young people in decision-making was apparent.^49^ Of course, this requires young people to articulate their preferences and this can be challenging and complex as competence and capacity will vary for each individual child.^55^ Professionals therefore need to think clearly about their role in this process. Young people report favourable experiences if trusting relationships are formed between the clinician and patient.^56^ Professionals who were confidential, did not withhold information and engaged in small talk to show concern are most likely to gain a young person's trust.^57^

Parents and/or guardians still have a role to play in shared decision-making for any healthcare decisions for a young person.^51^ Adults have reported they want to empower young people to be accountable for their own healthcare decisions;^54^ however, very rarely did they allow this to happen.^49^ Therefore, professionals need to actively encourage adults to allow children to express their opinions and preferences for decisions rather than dictating what they think they would want.^49^^,^^51^

## Management of cFPM

A full assessment of the developing dentition and the patient is required before presenting and discussing available treatment options for cFPMs. Several factors, including patients' oral health values and preferences, will influence these discussions.^10^^,^^25^^,^^49^^,^^54^ An overview of these factors can be seen in Table 1.

**Table 1:** Factors to be considered when treatment planning cFPMs

**Patient level**	**Mouth level**	**Tooth level**
Patient preferences	Number of affected cFPMs	Size and location of defect
Relevant medical history	Overall dental health	Number of surfaces involved
Age and level of co-operation	Dental developmental eg bifurcation of second permanent molars	Presence/absence of post-eruptive breakdown in hypomineralised tooth
Presence/absence of symptoms	Orthodontic need, such as presence/absence crowding, hypodontia etc	Pulpal involvement
Current access to general dental services	Presence of third permanent molars	History of dental abscess/facial cellulitis
Access to specialist care (paediatric dental/orthodontic)		

### Managing a cFPM with caries or MIH - does the condition matter?

Dental caries and MIH are not synonymous and thus pose different challenges when considering how best to manage cFPMs. In MIH, the FPMs are affected with the remaining posterior permanent teeth usually unaffected, potentially favouring a more extraction-based approach to management. In contrast, dental caries has the potential to affect all permanent teeth. Although, if patients and their parents engage with preventive strategies, then the disease burden could be minimal, potentially favouring a more restorative-based approach to management. Interestingly, recent qualitative evidence on managing cFPM due to caries and MIH with adolescents and adults would refute this theory, with restoration and retention of hypomineralised molars being preferred to elective removal, whereas this strong preference for a FPM compromised by dental caries was not apparent.^49^ Similar findings have been reported by dental professionals in both quantitative^58^^,^^59^ and qualitative^60^^,^^61^ studies, suggesting a paradigm shift in how patients want hypomineralised teeth to be managed.

Considering the treatment options for cFPMs, there are three general management strategies available:

- Active monitoring, accepting the tooth is likely to worsen without intervention
- Restoration, with the potential need for endodontic treatment
- Extraction, with potential for orthodontic or prosthetic treatment.

### Active monitoring

If the decision is to actively monitor a cFPM rather than restoring or extracting it, the risk of dental pain and subsequent infection and/or sepsis is high and should be discussed with the patient/parent.^19^^,^^40^ The carious lesion will progress, while the risk of further post-eruptive breakdown in an MIH-affected cFPM is likely. If the tooth remains untreated, any future treatment to deal with the broken/painful/abscessed cFPM would be more difficult, more expensive and have less certain results, with ultimately the only feasible option being removal.

### Restoration versus extraction

The key question of whether to restore or extract cFPMs in children remains unanswered.^10^^,^^49^^,^^50^^,^^62^ Given the advances in restorative materials and techniques,^63^ restoring is often a viable option for cFPMs with dental caries.

**Fig. 4:**
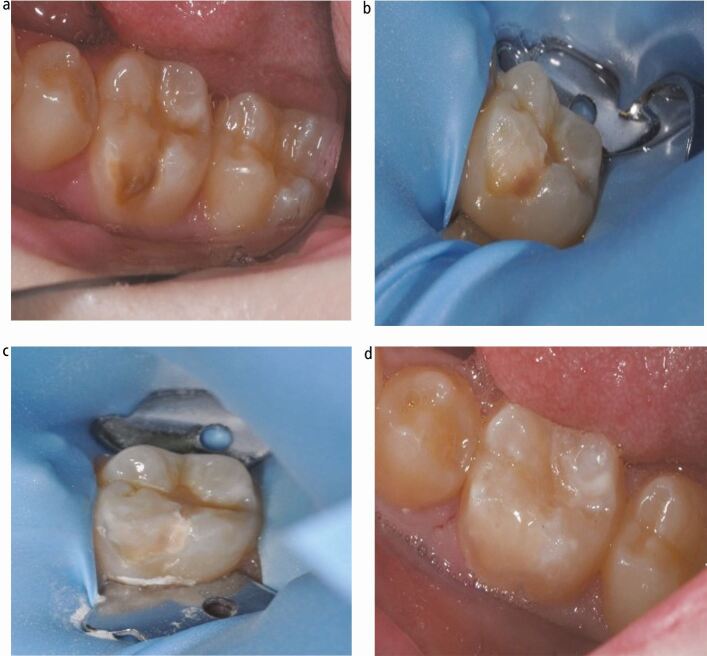
a, b, c, d) A hypomineralised lower left first molar (36) with post-eruptive breakdown (a) which has undergone partial hypomineralised enamel removal (b) and then complete hypomineralised enamel removal (c) leaving the margins of the restoration on sound enamel (d)

Historically, concerns were raised when restoring hypomineralised molars due to the weaker bond to hypomineralised enamel.^16^ Alterations in the microstructure and mineral content does mean that decreased bond strengths and higher failure rates, compared to sound enamel, are to be expected.^16^ However, despite a paucity of evidence, strategies such as extended etching and a pre-restoration rinse with sodium hypochlorite, which alters the protein-rich structures, have been suggested to improve adhesion and retention of composite to hypomineralised enamel.^41^^,^^64^^,^^65^ Regarding cavity design, total removal of all hypomineralised enamel has been suggested to mitigate against the known bonding issues of hypomineralised enamel.^10^ An alternative ‘selective' approach has been postulated, ensuring the cavity margins are on normal, sound enamel, and more hypomineralised enamel is left at the base. The latter reduces the risk of iatrogenic pulp exposure while, in theory, sufficiently mitigates against the bonding issues^10^ (see Fig. 4). These approaches appear to be promising in overcoming the structural issues with hypomineralised enamel; although, further research is required to provide a robust evidence base to support their regular adoption into routine practice.

If, during restoration, the pulp is exposed or the patient subsequently develops symptoms, endodontic strategies, such as vital pulp therapies, can be used to good effect for both carious and hypomineralised molars.^10^^,^^66^ Despite this, their use in young people with cFPMs in the UK is limited.^58^ Vital pulp therapies in cFPMs in young people have overall success rates of 91.3% (range: 78.5-100%) and 90.5% (range: 70-100%) for partial and coronal pulpotomies, respectively.^67^ However, if this approach is taken, it means entering the ‘restorative cycle' earlier in life, where any filling placed will eventually fail, requiring larger replacements each time, until such a point that there is no more tooth left to restore and the tooth is extracted.^68^

**Fig. 5:**
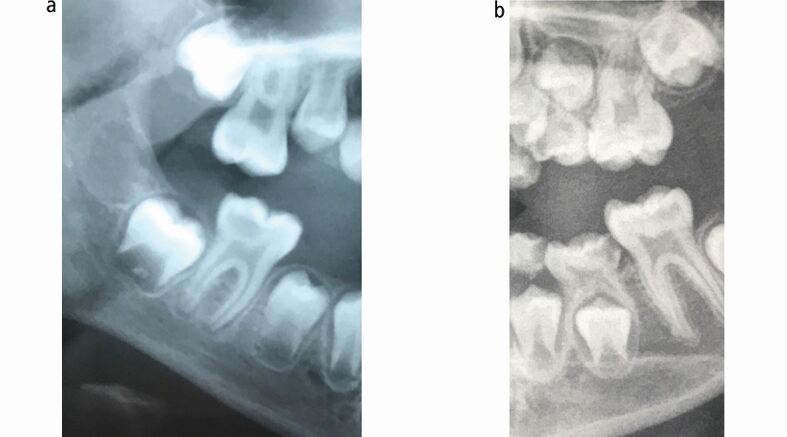
a, b) Key prognostic factors to support spontaneous closure: presence of third molar tooth; bifurcation calcifying of the second permanent molar; mesially angulated second permanent molar

The alternative - extraction - eliminates the need to maintain a restoration throughout life and for some patients/parents, this will be the preferred option.^49^^,^^68^ However, extraction is perceived by young people and adults to be more invasive than restoration, often cited by some as a reason to try and restore instead.^49^ Compared to a restoration, extraction of a cFPM in a young child will more regularly require the need for adjunctive treatments, such as sedation and general anaesthesia.^58^ The associated increased clinical risks and costs of these treatments need to be considered; however, in some clinical circumstances (eg severely affected hypomineralised cFPM with post-eruptive breakdown) extraction may be the only feasible option. If extraction is chosen, then spontaneous closure of the space, if carried out at the correct time, could occur, negating the need for orthodontic space closure or prosthetic replacement.^10^^,^^69^ Several radiographic prognostic factors (see Fig. 5), such as presence of the third permanent molar, mesial angulation and calcification of the bifurcation of the second permanent molar, if present, improve the chances of spontaneous closure following removal.^69^ However, even if all factors are present, spontaneous closure is not guaranteed and unfavourable tooth movement or tipping of adjacent teeth may occur.^70^

Consideration should be given to a compensating extraction of an upper FPM if the lower cFPM is to be extracted. However, this should be done with caution, and at least with a paediatric or orthodontic specialist opinion, as the recently updated national guidance now suggests that removal of a sound upper FPM should not be routinely carried out unless there is a clear occlusal requirement, or the upper FPM is going to be unopposed for a significant period of time.^69^

Removing cFPMs is likely to have an impact on the third permanent molar position. Despite a small sample size, a recent study demonstrated that the third molars of patients who had their cFPMs extracted between 8-11 years of age had moved significantly more mesially compared to the non-extraction control group. Theoretically, this could allow the third molar to erupt into a more favourable position and reduce impaction and associated morbidities that impacted third permanent molars can bring.^71^

Despite the advantages and disadvantages of these two main approaches, there is a paucity of clinical evidence that supports one approach over the other. Alternative methodologies have been used to overcome some of the uncertainty. Using a preference elicitation study, the UK public have no preference to restore or extract cFPMs in a young person, providing the resultant space was closed spontaneously or orthodontically.^54^ Similarly, a decision analytical model has been used to determine the incremental net benefit of initial options to manage cFPMs over a modelled lifetime horizon.^50^ The most efficient approach is to extract cFPMs between the age of 7-10, with definitively restoring still being an efficient option, but it is incrementally less cost-beneficial than extraction.^50^

## Conclusion

Preventing disease starting in FPMs should be a priority of dental professionals. However, should they become compromised, shared decisions actively involving young people need to be made.

## Data Availability

Not applicable.
